# Acceleration and Light-Induced Changes in Cytosolic cAMP Concentration in *Euglena gracilis*

**DOI:** 10.3390/biom16030451

**Published:** 2026-03-17

**Authors:** Peter Rolf Richter, Jenny Graf, Ferdinand W. M. Haag, Vanessa Scudlo, Selina Wiesmeth, Jens Hauslage, Martin Richter, David Geißler, Michael Lebert

**Affiliations:** 1 Gravitational Biology Group, Department of Biology, Friedrich-Alexander University, Staudtstraße 5, 91058 Erlangen, Germany; jenny.graf@med.ovgu.de (J.G.); ferdinand.haag@fau.de (F.W.M.H.); vanessa.scudlo@uk-erlangen.de (V.S.); david3.geissler@gmail.com (D.G.);; 2 Space Biology Unlimited, SAS, 24 Cours de l’Intendance, 33000 Bordeaux, France; 3 Institute of Aerospace Medicine, German Aerospace Centre, 51147 Cologne, Germany

**Keywords:** *Euglena gracilis*, photoactivated adenylate cyclase, cyclic AMP, phototaxis, gravitaxis, CRISPR Cas

## Abstract

The second messenger cyclic AMP (cAMP) is very likely involved in phototactic as well as gravitactic behavior of the unicellular flagellate *Euglena gracilis*. A slight but significant increase in cAMP was observed when cells encountered sub-threshold acceleration (0.16 × g) force after microgravity [µg]. No differences in cAMP levels were found between cells on a clinostat and 1x-controls. This observation is consistent with the ones of earlier studies. Illumination of cells resulted in a significant increase in cellular cAMP levels. After RNAi-mediated knockdown or CRISPR-Cas9 knockout of the photoactivated adenylyl cyclases PACα and/or PACβ in the photoreceptor, light-induced changes in cAMP levels were no longer observed. In parallel, phototactic behavior was abolished, supporting the essential role of photoactivated adenylyl cyclases in phototaxis. Cells spin around their length axis during locomotion (1–2 Hz). In order to generate a signal in the light direction, the cells should be capable of synthesizing and degrading cAMP within 0.5–1 s. The rapid fixation of cells upon transition from dark to light or light to dark revealed that detectable changes in cAMP-levels (increase or decrease) occur within a 100–200 ms time window, which is sufficiently fast to account for the proposed theoretical kinetics of cAMP oscillations.

## 1. Introduction

*Euglena gracilis* is a unicellular freshwater flagellate that has become an important organism for biotechnology and pharmaceuticals in recent years [1,2]. *Euglena gracilis* is one the fastest-growing representative of the *Euglena* genus and is considered highly promising due to its use in the so-called “5 Fs” (food, fibre, feed, fertilizer, fuel) [3,4]. Both the carbohydrate storage substance of *Euglena gracilis*, known as paramylon, and paramylon-free cell extracts have shown significant pharmacological effects, which are attributed, among other things, to the strong immunostimulatory properties of the cells [5,6,7,8,9].

The gravitaxis and phototaxis of *Euglena gracilis*, which mediate the orientation of cells in water [10], have been the subject of intensive research for many years [10]. According to a recent working model [10,11], gravitaxis is triggered when cells deviate from a vertical swimming direction. The entire cell content acts as a signal trigger by exerting gravitational force, which activates mechanosensitive elements, possibly calcium channels that are arranged in specific areas of the membrane [10]. This activation leads to a transient increase in intracellular calcium ion concentration, which is associated with a change in membrane potential [10,11,12]. Calcium ions activate a specific calmodulin 2 (CaM2 (EU935858), which, in turn, activates a as-yet-uncharacterized adenylate cyclase that synthesizes cAMP in its active state [10,11]. cAMP interacts with a specific protein kinase A (PKA: EU935859) [10,11], which influences flagellar beating in a manner that is not yet fully understood. This protein kinase was found to be located in the ampulla region of the cell and is also involved in the phototaxis of *Euglena gracilis* [13]. RNAi-mediated knockdown of the protein kinase leads to a loss of both gravitaxis and phototaxis and is accompanied by reduced transcription of the photoreceptor genes PACα and PACβ. Conversely, perturbation of the phototaxis pathway affects gravitactic behavior, indicating functional crosstalk between gravitaxis and phototaxis [13]. Additionally, a protein has been identified that interacts with CaM2 and plays a crucial role in gravitaxis (EgPCDUF4201) [14]. These new insights are based on the transcriptome and genome data from previous project phases [15].

Ozasa et al. 2019 investigated the photo-response of *Euglena gracilis* in combination with gravitaxis [16]. The researchers concluded that photo-shock (both step-up and step-down) can temporarily override gravitaxis, making cells sink even in the presence of a negative gravitational pull. The cells’ ability to adapt to light by switching from photo-shock responses to negative phototaxis demonstrates a dynamic mechanism of light adaptation. This ability allows Euglena to optimize its movement [17] to avoid damage from intense light while still responding to environmental cues like gravity and light direction. In addition, the study supports the view that *Euglena gracilis* actively controls its movement in response to gravity, relying on physiological processes that likely involve mechano-sensing and signaling proteins. The combination of active sensing and behavioral adjustment further reinforces the conclusion that gravitaxis in *Euglena* is an active, regulated physiological mechanism rather than a passive response.

Phototaxis is based on a blue light receptor located on the trailing flagellum of the cell, known as the PAB (paraxonemal body) [18]. The trailing flagellum emerges from an invagination at the front end of the cell, the reservoir or ampulla [19]. A second short flagellum does not leave the ampulla. There is still a debate about the nature of the photoreceptor of *Euglena gracilis*. The hypothesis that rhodopsin is the photoreceptor of *Euglena gracilis* is supported by various experimental proofs [20,21]. Microspectrophotometric analysis of the PAB revealed a photoreceptor photocycle characteristic of a rhodop-sin-based photoreceptor [22]. In a recent study of Lorenzetti et al. (2024), the photoreceptor of *Euglena gracilis* was investigated with Raman spectroscopy and compared with the Raman spectra of *Bos taurus* retina [23]. The spectra were very similar indicating strong evidence for rhodopsin presence in the PAB of *Euglena gracilis*. The spectra did not indicate the presence of flavin-mono-nucleotide FMN, which is the chromophoric group of the photoactivated adenyly cyclases, which are regarded to be the main photoreceptor molecules in another hypothesis. However, the FPLC analysis and the subsequent fluorescence spectroscopy of the separated proteins suggest the presence of flavins and pterins as chromophoric groups [24,25], and Iseki et al. (2002) purified flavoproteins from the PAB and published for the first time the protein sequence of PACα and PACβ [26]. This contradicts the statement of absence of flavin in the PAB. The knockdown or knockout of PACα or PACβ results in the loss of phototaxis, which very much supports their important role in phototaxis [26]. Brodhun and Häder (1995) [27] identified five major protein fractions from a desalted PFB-flagella extract, from which two showed pronounced flavin fluorescence and two showed pterin fluorescence. The presence of flavin and pterin fluorescence in the PAB was also described by Schmidt et al. (1990) by means of microspectrophotometry [28]. However, FPLC-analysis and subsequent fluorescence spectroscopy of the separated proteins suggest the presence of flavins and pterins as chromophoric groups [24,25] and Iseki et al. (2002) purified flavoproteins from the PAB and published the first time the protein sequence of PACα and PACβ [26]. In addition, the adenylyl cyclase activity was demonstrated with the isolated PAC molecules. In addition, up to now no significant rhodopsin or rhodopsin-related protein was found in the genome or transcriptome of *Euglena gracilis*. However, the elucidation of the role of flavin in PAC does not exclude a possible involvement of rhodopsin in photoperception, nor a potential interaction between rhodopsin and the PAC-mediated mechanism. For the interpretation of our data, we therefore refer exclusively to the flavin-based hypothesis.

The detailed analysis of PAC function above all in *Oscillatoria acuminata* revealed that upon bluelight absorption by the BLUF (Blue Light Using Flavin) domain, a key rearrangement occurs within the hydrogen bond network around the flavin chromophore [29,30,31]. This rearrangement involves residues such as Y6 and Q48, leading to a structural transition. Specifically, the light-induced activation triggers small changes in bond distances and angles, which, though minor at the molecular level, propagate through the protein structure. This structural shift in the BLUF domain activates the adenylate cyclase (AC) domain located in a distant part of the molecule. Flavin adenine dinucleotide (FAD) serves as the key chromophore. It undergoes a light-induced electron transition, leading to a red shift in its absorbance spectrum. This excited state of flavin is stabilized by hydrogen bond rearrangement in the BLUF domain, primarily around Y6 and Q48 [30]. The excitation of flavin upon absorbing blue light is the initial event in the cascade, causing structural and conformational changes in the PAC. Once flavin is excited by blue light, the structural rearrangement propagates from the BLUF domain to the AC domain via allosteric regulation. This process involves small conformational shifts in the linking helices and loops, leading to changes in the positioning of the catalytic sites of the AC domain. This propagation of structural change across the molecule is crucial for activating the adenylate cyclase domain. As the structural changes move through PAC, the AC domains undergo a significant conformational change, shifting from an inactive to an active state. These movements involve the opening of the catalytic cleft, which is essential for binding ATP. The activation of the AC domains results in their enhanced catalytic activity, with ATP binding and subsequent conversion to cAMP being initiated at this stage. Upon activation by light, ATP binds to the active site in the AC domain, which is formed by the dimer interface of PAC. The binding of ATP to this site involves coordination through divalent ions and specific residues that stabilize the ATP molecule. Structural studies revealed that the binding of ATP causes conformational changes in the PAC, expanding the enzyme and stabilizing its active form. The thermodynamic properties suggest that ATP binding is both entropy- and enthalpy-driven.

Recently, an interaction between phototaxis and gravitaxis was observed [13]. In addition to the synthesis of cAMP, the degradation of cAMP is also crucial for modulating the signal. This degradation is carried out by phosphodiesterases, which break down cAMP into AMP. The interplay between cAMP synthesis and degradation, regulated by the resulting cAMP levels, controls the previously mentioned protein kinase.

A drawback of using *Euglena gracilis* for biotechnological applications has been the lack of transformation methods. However, methods for Agrobacterium-mediated transformation and transformation using CRISPR-Cas9 [32] or Cas12a [33], respectively, have now been developed. A recently available chromosome-level de novo assembly of the genomic DNA of *Euglena gracilis* enables the identification of exon and intron sequences [34].

Employing RNA interference and CRISPR Cas9, mutants were generated in order to investigate the role of blue light-dependent adenylyl cyclases on the generation of cAMP [35,36]. In addition, this manuscript presents data of acceleration-dependent changes in cAMP production obtained on different µg platforms such as sounding rocket, parabolic flight, and clinorotation.

## 2. Materials and Methods

### 2.1. Cell Culture and Growth Condition

The fresh water flagellate *Euglena gracilis* Z KLEBS strain was obtained from the SAG, Göttingen [37]. Cells were cultivated under a constant illumination of 25 W/m^2^ of white light (a mixture of cold- and warm-white LEDs, respectively) at 20 °C. In order to keep constant growth conditions during campaigns such as parabolic flights or sounding rocket experiments, an isolated (Styrofoam plate foam insulation) culture box was built, which was equipped with identical LEDs providing the exact light conditions of the culture chamber. Cells were grown in a complex medium [38]. In order to achieve highly reproducible data, cells were incubated in 250 mL of medium in 500 mL Erlenmeyer flasks. Five million cells, determined with a Thoma chamber, were used as an inoculum. Twelve-to-sixteen-day-old cultures were employed for experiments after checking gravitactic and phototactic behavior by means of image analysis and the photometrical determination of the cell number (1.05 × 10^6^ cells/mL) by means of a suitable cell count formula, elaborated from the Thoma chamber-based cell counts. The formula for the determination of cell density is as follows:

*Cell count* [cells/mL] = (1.6342 × *OD*800 nm + 0.0926) × 10^6^

### 2.2. Fixation of Cells

Two methods were employed in order to rapidly fix the cells: (1) Fixation with double amount of MeOH: Cells were fixed by addition of a two-fold volume of methanol. Upon addition of methanol, cell motility ceased immediately, as verified by microscopic inspection, indicating rapid functional inactivation. After fixation, the cell–MeOH mixture was heated to 80 °C in order to inactivate any still active phosphodiesterase. Aliquots of the residue were evaporated with a vacuum concentrator. The residue was dissolved in 0.1 mol HCl. In order to not exceed the sensitivity range of the cAMP assay, not more than 350 µL of cell extract should be used at a cell density of the fixed culture of 10^6^ cells/mL. (2) Fixation with MeOH:chloroform 3:2 (*v*/*v*): Cells were fixed with a six-fold amount of MeOH:chloroform. A microscopic inspection showed the immediate cessation of motility and pronounced membrane damage, while the overall cellular outline remained visible and consistent with rapid membrane lysis and the preservation of cell shape by the pellicular layer. After fixation of protein, cell debris was precipitated overnight at −20 °C. The supernatant was evaporated by means of a vacuum concentrator, and the residue was dissolved in 0.1 mol HCl before being subjected to cAMP determination.

Due to technical constraints, events occurring within the first few hundred milli-seconds after fixative addition cannot be directly resolved. However, the rapid cessation of motility, microscopically observed membrane damage, and stable cAMP-levels indicate effective quenching within the temporal resolution of the method.

### 2.3. Image Analysis

Cell movement was recorded with the tracking software WinTrack2000 [39]. In this manuscript, the circular movement histograms are provided, which display the general movement of a cell population towards the vectors of light or acceleration, respectively. The “long-track” module of the software was employed to determine step-up and step-down photophobic reactions of the cells. Photophobic reactions are indicated by a sudden decrease in swimming velocity of 40% or spinning movements around the length axis for more than 350 ms. The program determines photophobic responses over time. The step-up photophobic response was elicited by exposing dark-adapted cells to blue light (peak wavelength 470 nm, photon fluence rate 71.4 µE). The step-down photophobic response was induced by switching off the blue-light source after the cells had adapted to the light regime. 

### 2.4. cAMP Determination

Intracellular cAMP was determined using the Direct cAMP ELISA kit (ADI-901-066) from Enzo Life Sciences, Inc. (Farmingdale, NY, USA) according to the supplier’s instructions (non-acetylated assay). In order to calculate the amount of cAMP per cell, the number of cells in a sample well of a 96-well plate was calculated from the determined cell number and the number of cells finally introduced into the sample wells of the microtiter plate. The measured cAMP concentrations of the sample wells were divided by the calculated cell numbers in order to get the number of cAMP molecules in attomoles (amol) per cell.

### 2.5. Induction of RNAi-PAC Alpha Knockdowns

The RNAi knockdown of PACα was performed as described earlier [40]. In brief, RNA was isolated from *Euglena gracilis* WT strains and transformed into cDNA. By means of PCR and suitable primers (forward primer: 5′ATGCTCAATATGAGCGACAATTTCGTGGAT3′, reverse primer: 5′ATGCGGGTGCCGTTCAGAAGGAT3′), an about 200 bp C-terminal sequence of the PACα gene was amplified by means of PCR. T7-promotor adapter was ligated to both ends of the PCR product. The T7-promotor adapter was produced by a combination of the following DNA-oligos: 5′TAGCGTAATACGACTCACTATAGGGT3′, which was additionally used as a PCR primer later, and 5′-PHO-CCCTATAGTGAGTCGTATTACGCTATG3′ (5′-end phosphorylated to enable ligation to the PCR product). The oligos were heated for 5 min to 80 °C. Subsequent slow cooling to room temperature for about 1 h resulted in the formation of T7-promotor adapter. The ligation product was amplified using the abovementioned T7-oligo as a primer and thereafter translated into dsRNA using the in vitro transcription MEGAscript™ T7 kit (Invitrogen, Thermo Fisher Scientific, Carlsbad, CA, USA) according to the manufacturer’s instruction.

### 2.6. Production of Photoreceptor Mutants by Means of CRISPR Cas9

CRISPR-Cas9 knockouts were generated following the protocol described by Nomura et al. (2020) [41]. In contrast to the procedure described in Nomura et al., cells were grown in EM medium. In brief, Cas9 ribonucleoproteins consisting of crRNA, tracrRNA, and Cas9 endonuclease were introduced into cells by means of electroporation. In order to hybridize crRNA and tracrRNA, each 0.6 µL of 100 µM solutions were mixed and heated (95 °C for 5 min). After cooling to room temperature, 0.8 µL of Cas9 enzyme (Alt-R S.p. Cas9 Nucleoase V3 (IDT, 62 µM)) was added to each crRNA-tracrRNA complex. About 10^6^ *Euglena gracilis* cells were washed and resuspended in an electroporation solution (CM medium and 0.3 M sucrose mixed in a ratio 3:2 *v*/*v*). Electroporation was performed in 2 mm cuvettes using a NEPA21 Super Electroporator (Nepagene Co., Ltd., Chiba, Japan). Setting of the electroporator was as follows: 2 poring pulses: 300 V, pulse length 5 ms, pulse interval 50 ms, decay rate 40%, polarity switching +; 5 transfer pulses: voltage 20 V, pulse length 50 ms, decay rate 40%, polarity switch −/+. After electroporation, the cells were incubated in EM supplemented with 0.3 M sucrose (ratio 99 in 1 *v*/*v*) in darkness for two days. Cells without previous mixing served as controls. Small aliquots of the preculture (approximately 10 µL) were then transferred into the first row of wells in 48-well plates and serially diluted 1:1 with EM medium across seven dilution steps. After approximately 10 days, the plates were examined for cell growth. In the later dilution steps, no cells were observed, indicating that the culture in the last well showing growth was likely derived from only a few individual cells. This effectively reduced the heterogeneity of the original electroporated cell culture. The phototactic behavior of the cells from the final wells was assessed. Cultures displaying impaired phototaxis were plated onto EM agar plates containing 2 g/L sucrose and 1 µg/mL ampicillin to prevent contamination. The plates were sealed with micropore tape and incubated one day in the dark at 20 °C. Subsequently, cells were exposed to light at 25 W/m^2^ and incubated at 28 °C for about 5 days. Single colonies were then isolated from these plates and subsequently cultured in EM medium. The cDNA of stable transformants was sequenced and analyzed in order to verify the effect of CRISPR Cas9 treatment.

The used crRNA sequences are shown in Table 1. Single knockouts of wildtype cells by means of these crRNA sequences were produced. In addition, double mutants were established by the knockout of PACβ in an already PACα-deficient mutant (Klone E).

### 2.7. Parabolic Flight Campaign

To investigate the signaling pathways for light (phototaxis) and gravity (gravitaxis) in *Euglena gracilis* and explore a possible interaction between them, an experiment was conducted during the 41st DLR parabolic flight campaign, collecting 216 samples over three flight days. The experiment setup included a scaffold with three boxes, each holding 24 samples. Each box was connected to the aircraft’s power supply, and the samples were exposed to different light durations during parabolic flight. A syringe system with motors controlled the fixation of cells with methanol/chloroform during the flight. Samples were fixed in three phases of each parabola, i.e., hypergravity (1.8 g), microgravity (0 g), and normal gravity (1 g), with switches manually triggered to activate the motors.

Before each flight, Euglena cells were cultured in EM medium with ampicillin for nine days under light at 20 °C. During the flights, their phototactic and gravitactic behavior was recorded using image processing systems (ECOTOX, Wintrack1000). Additionally, Optical Density (OD) at 800 nm was measured for later analysis.

In-flight sampling: During the flight, each parabola was divided into phases. At specific moments (e.g., just before hypergravity or during microgravity), switches were triggered to fix the samples in methanol/chloroform using pre-programmed motors. Due to technical limitations, not all boxes could be triggered simultaneously.

Post-flight Processing: After the flight, samples were processed by evaporating the liquid, heating, and centrifuging. Methanol was added, followed by further centrifugation. Prepared samples were stored at 4 °C until analysis using a cAMP ELISA kit (2.4). Cell counts were calculated based on OD_800_ measurements, following the established formulas.

### 2.8. Fast Fixation of Cells for Determiantion of Light-Dependent cAMP Kinetics

A fully automated hardware and software system was developed that rapidly mixes fixation medium with cell suspensions using paired syringes. Up to six syringe pairs can be fixed simultaneously. The syringe pistons are actuated by four parallel linear solenoid electromagnets, allowing fixation within less than 10–20 ms. Controllable LEDs are positioned directly next to the cell-containing syringes to provide defined light stimuli. Piston movements are monitored using reed switches. The fixation process is controlled by an Arduino Mega 2560. Timing intervals for LED activation can be freely configured. Switching events and sensor signals are transmitted to a computer via an RS232 interface, allowing a precise correlation between illumination profiles and fixation time points. Two operation modes were implemented:

(1) Cells are fixed after different periods of illumination (initial dark phase followed by light exposure) to determine the activity of the blue light-activated adenylate cyclase.
(2) Illumination starts at the beginning and is then switched off sequentially for each sample, resulting in defined dark periods. This mode is used to determine phosphodiesterase activity (cAMP degradation in darkness).

### 2.9. Sounding Rocket Experiment on MAXUS

The module was designed by the TSID3 (TEXUS/MAXUS) department at Airbus Defence and Space (Bremen, Germany). It consisted of two identical centrifuge platforms, each with two fixation chambers and two observation units. The platforms spun in opposite directions to compensate for angular momentum, allowing for defined sub-1 g accelerations, with all cells experiencing the same acceleration.

Observation units were equipped with infrared diodes for video recording, a mirror and digital camera (frame rate 30 Hz), and two motors to control the focus and movement of the cuvette. Two of the four units had blue light LEDs to expose the cells to light–dark cycles.

Fixation chambers held 30 syringe sets arranged in six rows of 5. Three rows had blue light LEDs to expose the cells to light phases. The syringe sets contained 0.25 mL of cells and 1.25 mL of fixative (methanol–chloroform), which were connected. A pneumatic system applied pressure, pushing the fixative into the syringe containing the cells.

Samples were fixed according to a schedule, with duplicates under both dark and light conditions. There were 30 fixation steps, with a total 120 samples. The samples were used for RNA isolation, protein isolation, or cAMP concentration measurements.

Cultivation and Integration into the Rocket: *Euglena gracilis* was cultured in 1000 mL Erlenmeyer flasks with 500 mL of EM medium. After 8 days, gravitaxis was measured, and the cell count was determined. The cells were concentrated to 4 million cells/mL, loaded into syringes, and stored under optimal conditions until integrated into the fixation chambers. Integration into the rocket took about 3.5 h.

Storage and Isolation: After the payload was returned, the cells were fixed at −20 °C and processed for cAMP and protein analysis. The samples were prepared for SDS-PAGE or mass spectrometry and stored at −80 °C.

### 2.10. Clinostat Experiments

Clinostat experiments were performed with a pipette-clinostat, which is described elsewhere [42]. After the determination of cell number, samples were filled in 1 mL pipettes and placed horizontally on the clinostat. For cell fixation, all pipettes were tilted simultaneously transferring the cell into 2 mL of EtOH. Subsequently, cAMP was determined by means of an ELISA assay.

## 3. Results

### 3.1. Changes in Intracellular cAMP Concentration at Different Sub-1 gAccelerations and Irradiations (Results of MAXUS 9)

**Figure 1:**
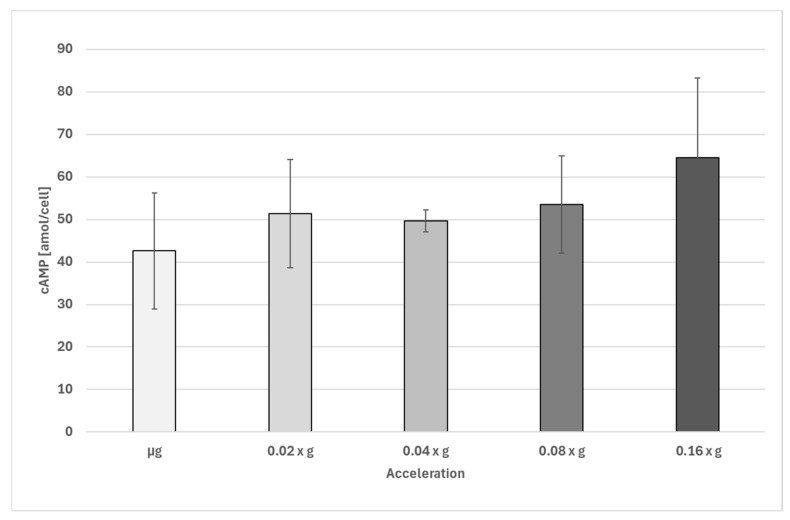
Intracellular cAMP concentration (amol/cell) in *Euglena gracilis* at different sub-1 g accelerations determined during a sounding rocket experiment on MAXUS 9. Number of biological replicates, µg: n = 14; all other groups, n = 5. The error bars show the standard deviation. Only the difference between µg and 0.16 × g is significant (*p* < 0.05).

Dark-incubated samples showed a slight increase in cAMP levels upon increasing acceleration (Figure 1). However, due to the high variation in the data, only the difference between µg and 0.16 × g was found to be significant. The increase in cAMP was about 66%.

### 3.2. Effect of Clinorotation on Intracellular cAMP Levels

**Figure 2:**
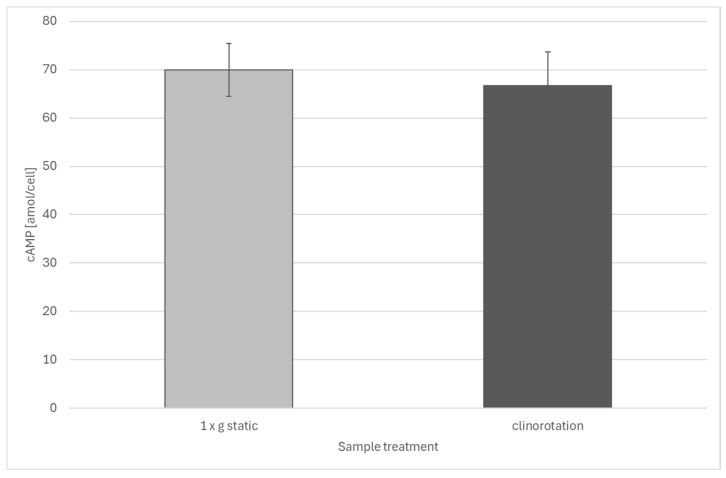
Cyclic AMP levels of stationary and clinorotated *Euglena gracilis* cells. Replicates: 10 biological replicates with 2 technical replicates each. The error bars show the standard deviation. There are no significant differences between the two groups.

No significant changes in intracellular cAMP levels were detected in cells during clinorotation, compared to stationary cell samples (Figure 2).

### 3.3. Time-Dependent, Light- or Dark-Dependent Increase or Decrease in cAMP

**Figure 3:**
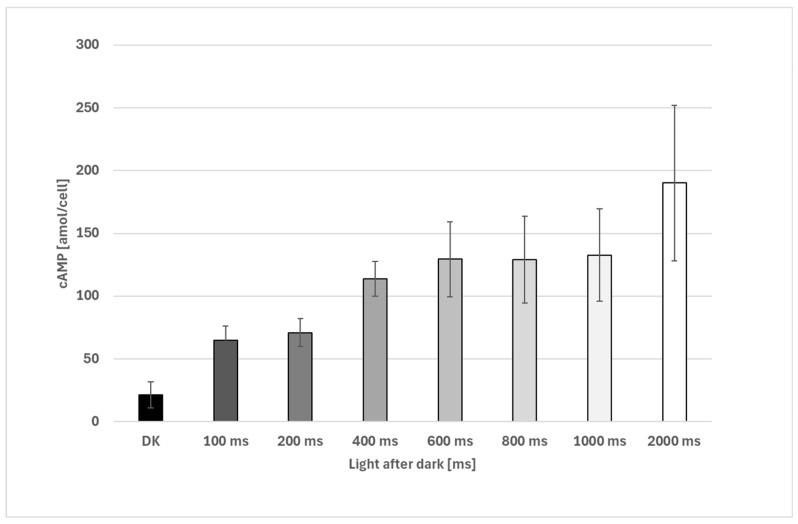
Increase in cAMP after illumination of dark-incubated cells. Samples were taken with an automated sampling device. All illuminated samples exhibit significantly higher cAMP levels compared to the controls (100 ms and 2000 ms, respectively, *p* < 0.05, all other groups, *p* < 0.05, Welch’s two-sample *t*-test). Replicates: 5 biological replicates, 2000 ms, only 4 biological replicates, with 2 technical replicates for each measurement. The error bars show the standard deviation.

Samples were collected by means of an automated sample device, which enables short-term illumination times and subsequent rapid fixation. Dark-incubated cells showed a fast increase in cAMP levels upon illumination (cold-white LED light, peak 450 nm, ≥100 μE). After 100 ms, intracellular cAMP levels increased significantly from about 21 amol/cell to about 65 amol and further increased to a level of about 130 amol/cell after one second (Figure 3). Rapid fixation resulted in the immediate loss of motility and microscopically evident membrane damage, while overall cell shape was preserved, consistent with fast enzymatic inactivation under the applied conditions.

**Figure 4:**
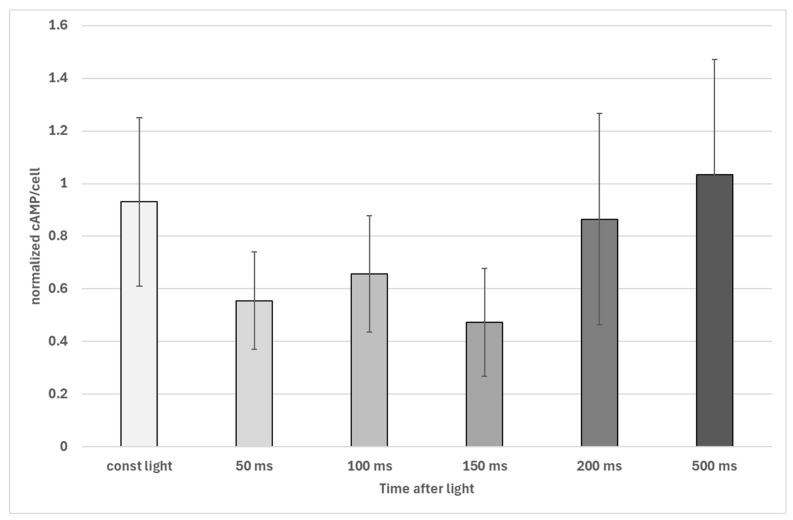
Decrease in intracellular cAMP levels in *Euglena gracilis* after exposure to darkness after illumination. Time caption indicated time in darkness after previous illumination. Data were collected with an automated sampling device. The figure shows data of four different experiments, where all data were normalized to the corresponding average of the light-exposed samples by dividing all particular cAMP levels by the average. All normalized data of a treatment were pooled and are presented with their standard deviation. The decrease after 50 ms, 100 ms, and 150 ms, respectively, is significant (*p* < 0.01, Welch’s two-sample *t*-test), and normalized cAMP averages after 200 ms und 500 ms, respectively, are not significantly different from those of the control.

The sudden switch of light was followed by a sudden decrease in intracellular cAMP concentrations in *Euglena gracilis* cells. After about 50 ms, a significant decrease in cAMP concentration was observed, which lasted until 150 ms (Figure 4). After 200 ms in darkness, cAMP levels show an increase, an observation that needs further investigation. 

**Figure 5:**
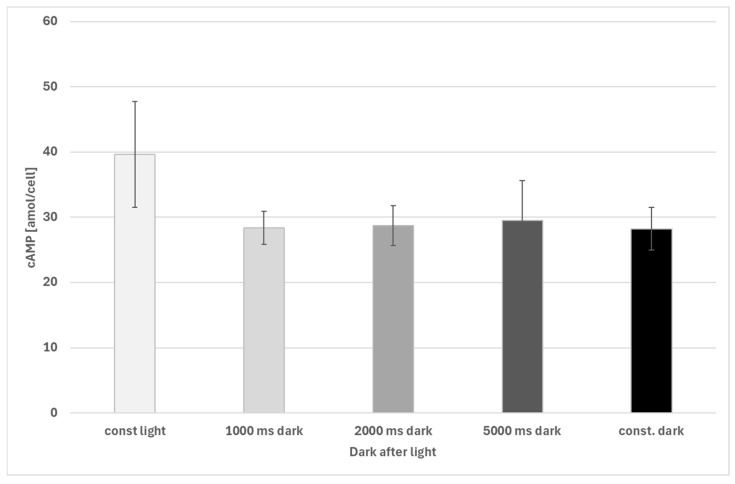
Decrease in intracellular cAMP levels in *Euglena gracilis* after exposure to darkness after illumination. Time caption indicated time in darkness after previous illumination. Differences between samples and cells kept under constant light (const light) are significant (1000 ms, 2000 ms and dark control (const. dark): *p* < 0.001; 5000 ms in darkness after light: *p* < 0.05, Welch’s two-sample *t*-test). Changes in cAMP in cells after illumination is not significantly different from cells kept in constant darkness (const dark). Replicates: const dark: 17 biological replicates; other groups: 8 biological replicates. The error bars show the standard deviation.

After dark incubations of more than 1 s, detected cAMP levels are in the range of dark-incubated cells (>30 min in darkness) (Figure 5).

### 3.4. Results of cAMP Change Obtained During Parabolic Flights

**Figure 6:**
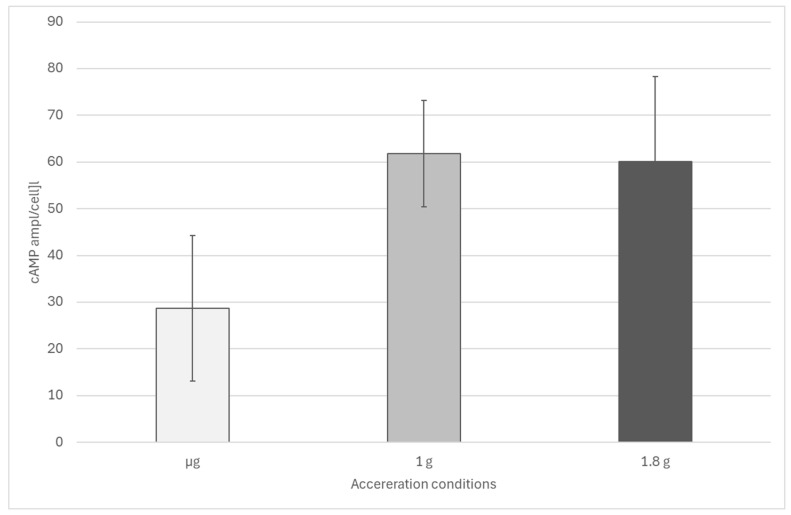
Effect of acceleration on cellular cAMP levels in *Euglena gracilis* obtained during a parabolic flight. Difference between µg (n = 8) and accelerated samples is significant (*p* < 0.01, Welch’s two-sample *t*-test). Difference between 1 g (n = 8) and 1.8 g (n = 17) is not significant. The error bars show the standard deviation.

During parabolic flights, slight but significant differences between µg samples and samples fixated under acceleration conditions were observed (Figure 6). While cells under microgravity showed an average cAMP level of about 34 amol/cell (with strong variations), in accelerated samples, cAMP levels of about 65 amol/cell were detected. No significant differences were detected between samples exposed to 1 × g or 1.8 × g.

**Figure 7:**
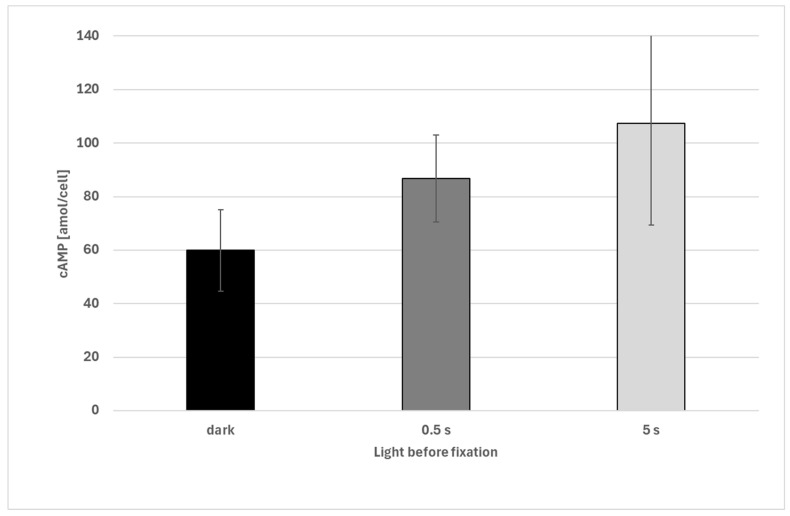
Effect of light on cAMP levels in *Euglena gracilis* cells. All dark data were pooled. Differences between dark-incubated cells (n = 30) and illuminated samples are highly significant (*p* < 0.01, Welch’s two-sample *t*-test). Difference between 0.5 s (n = 14) and 5 s (n = 17) are significant (*p* < 0.05). The error bars show the standard deviation.

Cells exposed to light showed a significant increase in cAMP after 0.5 s of illumination. When all dark-exposed samples of all acceleration steps were pooled, average cAMP levels were found to increase from about 34 amol/cell (darkness) to about 87 amol/cell after 0.5 s or 107 amol/cell after about 5 s (Figure 7).

**Figure 8:**
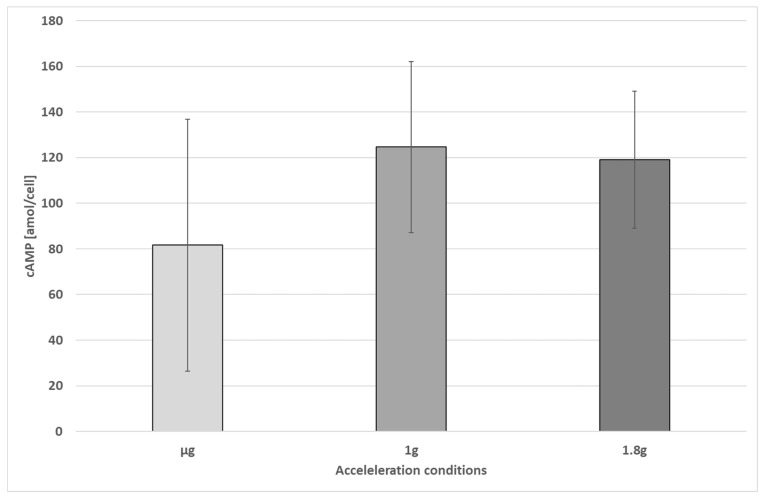
Cyclic AMP levels of illuminated cells under different acceleration conditions (no sig. changes, Welch’s two-sample *t*-test). The error bars show the standard deviation.

No significant differences were found in illuminated cells at different acceleration phases (Figure 8). However, in all illuminated cells, high data scattering was observed.

**Figure 9:**
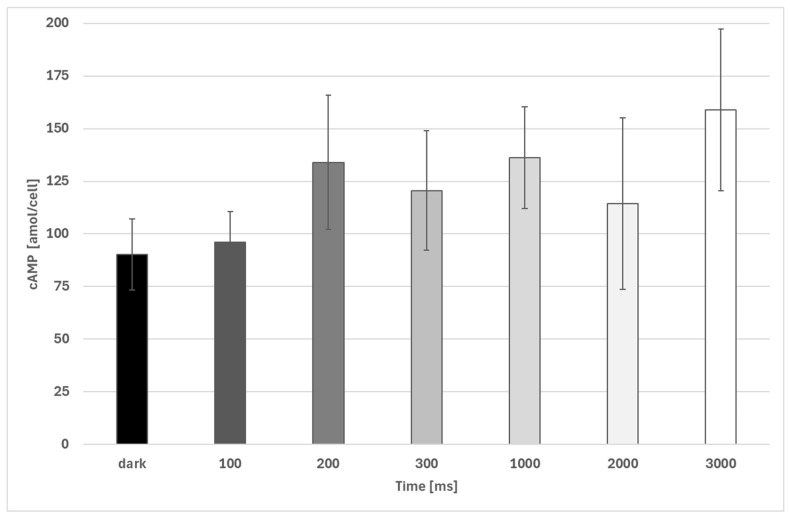
Changes in cellular cAMP concentration in *Euglena gracilis* cells in correlation to irradiation time. Data were obtained during a parabolic flight campaign. n: dark control: 18, with two technical replicates each; irradiated samples: 9, with 2 technical replicates each. All light-exposed cells show significantly higher cAMP levels except at 100 ms and 2000 ms (high data deviation) (Welch’s two-sample *t*-test). The error bars show the standard deviation.

Dependencies of cAMP and illumination time were measured in a parabolic flight campaign (DLR-PFC41, September 2023). A significant increase in cAMP was determined after 200 ms of illumination compared to the dark controls (Figure 9). The initial cAMP concentrations increased from 80 amol/cell to 103 amol/cell after 200 ms. The increase after 100 ms was not significant compared to the dark control. Subsequently, all illuminated cells exhibited significantly higher cAMP concentrations (except after 2000 ms, due to high variations in the data).

### 3.5. Effect of RNAi Knockdown on Phototaxis and Light-Induced cAMP Changes

**Figure 10:**
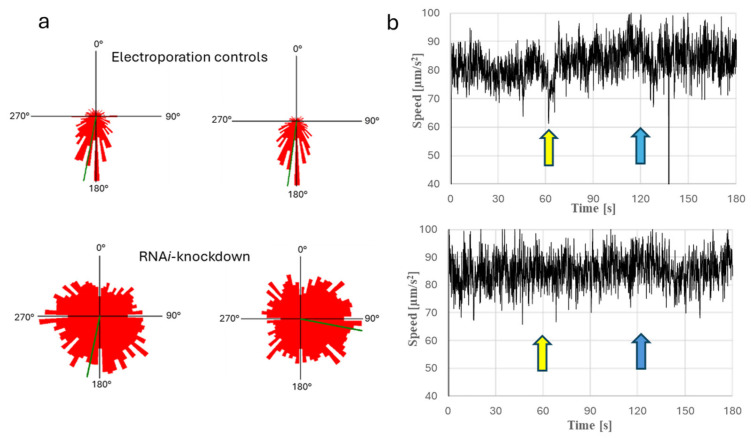
(**a**): Histograms of phototactic movement of RNAi-knockdown clones or control cells. The controls in both groups exhibit directional movement away from the light source for phototaxis, whereas the RNAi-knockdown cells do not show a special directional behavior, meaning the cells move randomly in every direction with Pacα knockdown. Cells were irradiated from 0°. The length of the red sectors shows the fraction of cell movement in the corresponding direction. (**b**): Diagrams in the right show the step-up and step-down photophobic responses in *Euglena gracilis*; left figure: control cellsl right figure: mutants after RNAi knockdown of PACα. Cells were incubated in darkness 5 min before measurement and before recording of cell movement was started. After 60 s in darkness, light (blue light, peak 475 nm, 74.15 µE) was switched on for 120 s (induction of step-up photophobic response). At 120 s, light was switched off again (induction of step-down photophobic response). Data show the mean velocity of recorded cells at a certain time point. Arrows indicate phobic changes in swimming speed. The light arrow indicates the step-up photophobic response, and the dark arrow indicates the step-down photophobic response.

After the RNAi knockdown of PACα, *Euglena gracilis* cells did not show negative phototaxis, which in turn was very pronounced in the electroporation control (Figure 10a). While control cells exhibit step-up and step-down photophobic responses, cells with RNAi knockdown of PACα did not show a step-up photophobic response and showed a weak step-down photophobic response (Figure 10b).

**Figure 11:**
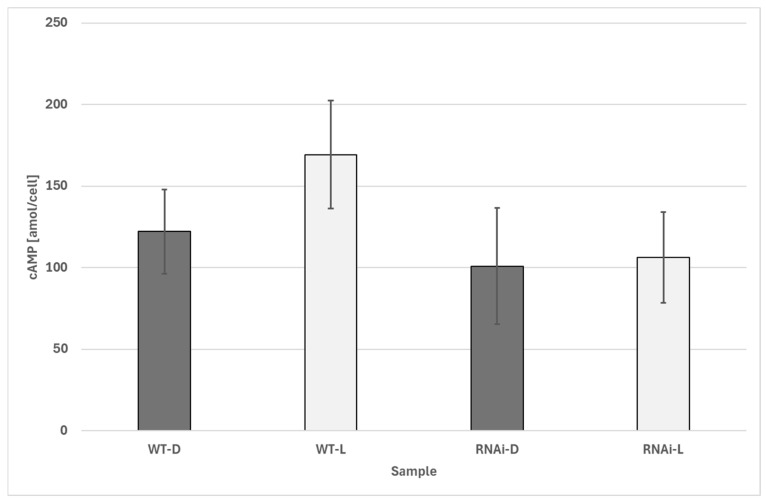
Changes in cellular cAMP concentration in *Euglena gracilis* cells upon illumination. WT-D: dark control; WT-H: illuminated control; RNAi-D: Euglena gracilis cells after RNAi knockdown of PAC in darkness; RNAi-L: *Euglena gracilis* cells after RNAi knockdown of PACα after illumination. n: control cells (dark and light, respectively): each data point consists of at least 27 independent samples. Differences between WT dark and WT illuminated are significant (*p* < 0.01, Welch’s two-sample *t*-test). No significant differences between dark-incubated and illuminated RNAi knockdowns. The error bars show the standard deviation.

In light, changes in light-dependent cAMP synthesis were significant in electroporated (without dsRNA) wildtype cells, while cells after RNAi knockdown of PACα did not show significant differences in their cAMP levels in both darkness and light (Figure 11).

### 3.6. Effect of Light on cAMP Synthesis in CRISPR Cas9 Mutants of PACα and PACβ or Their Double Mutants

**Figure 12:**
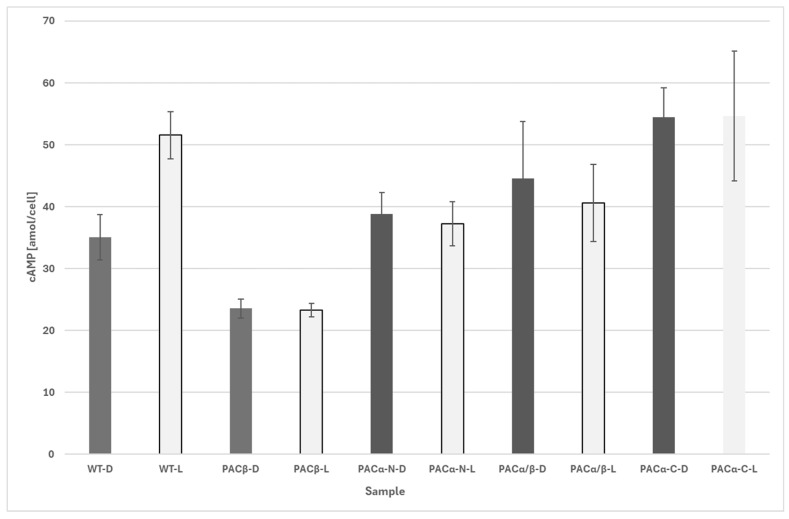
Effect of light on cAMP levels in *Euglena gracilis* mutants. Not illuminated (“-D”) vs. illuminated (“-L”) samples. WT: wildtype cells; PACβ: PACβ-knockout mutants; PACα-N: PACα-knockout mutants (deletion at N-terminus); PACα/β: knockout mutants; PACα-C:_KO: knockout mutants with knockout at the C-terminus of PACα. Each data point consists of eight independent biological samples. Only the difference between WT-D and WT-L is significant (*p* < 0.01, Welch’s two-sample *t*-test). No significant changes were observed between a mutant in darkness and after illumination. The error bars show the standard deviation.

Different CRISPR Cas9-knockouts, such as PACα-KO, PACβ-KO, double KO of PACα and PACβ, and the knockdown of the C-terminus of PACα, were investigated. None of the mutant strains exhibited phototaxis, while the control cells showed precise negative phototactic behavior at lateral illumination with blue light (peak wavelength: 475 nm) of 74.13 µE. While control cells showed significant increase in cAMP after illumination, no significant changes in cAMP levels were detected in the mutant strains before and after illumination (Figure 12).

## 4. Discussion

### 4.1. Acceleration-Dependent Changes in Cellular cAMP Levels

In parabolic flight experiments as well as during the sounding rocket experiment on MAXUS, a slight but significant increase in cAMP was determined from µg to acceleration. During the sounding rocket experiments, an increase in cAMP was observed at 0.16 *×* g acceleration. This confirms the inference of previous reports that microgravity affects cellular cAMP levels [43], where a significant increase in cAMP was already observed after 3 s of acceleration at 0.08 × g. The observed cellular cAMP concentrations (about 40–100 amol per cell, depending on acceleration) determined in this study are comparable with the values obtained by Tahedl et al., which were detected by means of a different cAMP ELISA assay. The observations coincide with those of studies where the threshold of gravitaxis was determined to be 0.12 *×* g [11]. As no gravitaxis was observed at accelerations below 0.12 *×* g, it is likely that this threshold is based on physiological perception and signal transduction mechanisms because passive alignment would also be visible at lower accelerations. Also, under the conditions of parabolic flights, lower cAMP levels were measured in µg, which indicated mechanosensitive events triggering cAMP evolution under acceleration conditions. No changes in cAMP were observed under clinorotation. Although the pipette-clinostat provides an almost shear force-free environment [44], clinorotation is not a measure to abolish effects of the gravity vector, which continue acting on cellular mechanosensitive elements and, above all, when cells are constantly moved with respect to the acceleration vector. It is proposed that mechanosensitive elements are most likely inserted asymmetrically in certain areas of the membrane, most likely at one defined membrane patch close to the reservoir. These elements are likely mechanosensitive membrane channels, which are activated by the sedimentation of cell body when the sensitive membrane patch is directed downwards. When cells swim parallel to the gravity vector, these elements are not triggered by gravity. However, when cells deviate from vertical alignment, these elements interfere with the acceleration vector. The flagellar beat frequency of *Euglena gracilis* cells is between 20 and 40 Hz. The flagellar stroke not only propulses the cells with an average speed of 50–100 µm/s but also makes the cells rotate counterclockwise around their length axis with a frequency of about 1–2 Hz [45]. It is crucial for the hypothesis of gravitaxis that the signal is produced and eliminated within the timeframe of one cell rotation, allowing correct reorientation strokes in the course of a spin. It still needs to be found out whether the time constant for the generation of signals (above all, Ca^2+^ and cAMP) is fast enough to react within the range of half the time of a cell rotation, in order to allow the adequate reorientation strokes of the flagellum. This can be achieved with a modified fast fixation device as used for the determination of light-dependent cAMP increase.

### 4.2. Light-Dependent Changes in Cellular cAMP Levels

Although the capacity of light-induced cAMP production of PAC was found in vitro, this is the first study where light-induced synthesis and subsequent cAMP degradation in the darkness were investigated. According to the flavin hypothesis, the photoreceptor consists of quasi-crystalline arranged flavin-binding receptor proteins, each composed of homodimers of two subunits, PACα and PACβ, which are activated by blue light absorption and synthesize cAMP from ATP [26]. Phototaxis is triggered by blue light-activated adenylate cyclase, which synthesizes cAMP upon irradiation [26]. The crystalline arrangement of flavin molecules in the PAB of *Euglena gracilis* ensures that the chromophores align similarly to the light vector. Experiments have proved the presence of dichroic orientation in Euglena’s photoreceptor molecules, where the photoreceptive pigments align with the electric dipole moment of the polarized light [46]. This causes maximal absorption when the flavins are perpendicular to the light and minimal absorption when they are parallel. As *Euglena gracilis* cells rotate around their longitudinal axis during locomotion, lateral illumination causes the dichroic photoreceptor absorption plane to align perpendicular to the light vector twice per rotation, resulting in two absorption maxima. Suppressing one of the two maxima, *Euglena gracilis* possesses a structure, the so-called eyespot or stigma. The stigma is composed out of vesicles (each about 0.4 µm) filled with carotenoids [47,48]. The absorption spectrum of the stigma overlaps with that of the photoreceptor, allowing the stigma to shield the receptor from blue light when positioned between it and the light source. Located at the level of the ampulla and the photoreceptor, the stigma is situated asymmetrically on one side of the cell. As the cell rotates, this arrangement ensures that the stigma casts its shadow over the photoreceptor once per rotation when light enters from the side. Experiments with roseoflavin shifted the absorption range, causing two maxima during rotation, revealing biphasic phototactic behavior [49]. Due to the dichroic arrangement of the photoreceptor molecules, the cells can not only determine light intensity but also light direction. At low irradiation, the cells swim toward the light source (positive phototaxis), while at higher irradiation, they swim away from it (negative phototaxis) [11,50]. However, it was shown recently that the stigma, which is regarded solely as a shading structure, may also play an important role in phototaxis as experiments with defective stigma (CRISPR Cas9 knockouts of lycopene cyclase or double mutant of β-Carotene Hydroxylase) have revealed, because the lack of zeaxanthin results in a loss of phototactic behavior [51].The authors speculate that zeaxanthin may play a role in the photoprotective mechanisms of the eyespot, protecting it from light-induced damage through its antioxidant properties.

Although sub-second events cannot be directly visualized, the immediate cessation of motility, microscopically evident membrane damage, and long-term stability of cAMP strongly support the rapid quenching of enzymatic activity in the fast-fixation protocol. The experiments in this study where the fast fixation of cells in light after darkness or cells in darkness after light was performed have shown that the generation of a modulated cAMP can be achieved in the timeframe of a cell rotation. The necessary time in order to increase or decrease the cellular cAMP concentration is around 200 ms. This kinetic is fast enough to allow the synthesis and degradation of cAMP during one cell rotation, which is a prerequisite of the PAC hypothesis. In experiments in which PAC was expressed in Xenopus oocytes to manipulate intracellular cAMP levels for the investigation of the cystic fibrosis transmembrane conductance regulator (CFTR), Schröder et al. estimated the activation time constant of PACα to be below 20 ms. This value is in good agreement with the rapid cellular responses observed in our study [52].

The prominent role of PACα and PACβ in light-dependent cAMP synthesis is clearly demonstrated by the experiments with PAC mutants (RNAi-knockdown and CRISPR Cas9-knockdown). In these mutants, phototaxis and the ability of light-induced cAMP synthesis are absent. One the other hand, the mutant experiments clearly prove that only the photoreceptor molecules but no other processes such as photosynthesis are responsible for the observed light-induced increase in cAMP.

### 4.3. Effect of Gravity and Light on cAMP Levels

No significant differences were found in cells that were irradiated under µg and accelerated conditions. As the light-induced increase in cAMP in general is far more pronounced compared to the acceleration-dependent increase and the data deviation is considerably pronounced, a clear inference about the interplay of light perception and acceleration perception has not yet been drawn from the results and probably deserves further research.

Although no synergistic changes in cAMP levels were detected when combining light and acceleration stimuli, several lines of evidence indicate that both sensory pathways are linked at the molecular level. A previous work demonstrated that RNAi-mediated downregulation of PACα causes the loss of both phototaxis and gravitaxis [13], suggesting that cAMP acts as a common second messenger integrating light- and gravity-dependent signaling. The Ca^2+^/calmodulin-dependent activation of adenylate cyclase during gravitaxis [22,23] and the light-induced activation of PACα/β both converge on cAMP production, which, in turn, modulates flagellar motility through PKA [24]. The absence of clear additive effects in the current experiments could therefore reflect dominant signaling by one pathway, different activation thresholds, or temporal desynchronization between the two stimuli. It is also possible that mechanical and photic inputs are processed sequentially rather than simultaneously, ensuring stable orientation under changing environmental conditions. Future studies combining the higher temporal resolution of cAMP dynamics with targeted mutants in both pathways will help to clarify how light and gravity cues are integrated at the signaling level. Accordingly, the acceleration-dependent results are presented as descriptive observations rather than a fully resolved mechanistic framework.

### 4.4. Possible Effects on Flagellar Stroke and Estimations

Tsang et al. (2018) have carefully investigated movement of *Euglena gracilis* cells under low-light and high-light conditions [45]. The authors documented helical swimming behavior under low-light conditions. After an increase in light intensity, cells started to show alternate spinning movement and helical movement, resulting in a polygonal swimming path. Further analysis revealed changes in the flagellar beating pattern. While during helical swimming “the flagellum twists into two loops that are distributed on both sides of the cell”, the flagella is directed to the front forming only one loop. The observed polygonal swimming pattern is due to the alternating shifts in the flagellum between the two stages. Similar behavior was observed during parabolic flights, where the flagellum of *Euglena gracilis* cells was pointed forward and formed one specific loop in microgravity [53]. This shows that both light- and acceleration-induced changes generate physiological signals, which regulate the flagellar beating pattern, resulting in changes in movement direction. The observed “spinning lasso” movement of the flagellum is probably caused by the paraflagellar rod (PFR), a protein rod aligned to and attached to the axoneme [29]. The attachment of the PFR with the axoneme created a structural asymmetry. Both the axoneme, which generates the beating motion via dynein motor activity, and the PFR, which is considered passive, cannot reach their respective energy minima simultaneously due to their structural incompatibility. The result is an “elastically frustrated” system, where the axoneme and the PFR push and pull each other, leading to out-of-plane deformations. The model predicts that this structural antagonism results in the torsional waves characteristic of the spinning lasso, producing non-planar flagellar shapes. The model is tested using hydrodynamic simulations that show how the interaction between the axoneme and the PFR leads to the generation of torsional peaks with alternating signs, matching the experimental observations of Euglena flagellar behavior. This non-planar beat is shown to be necessary for Euglena’s phototactic behavior, where the cell adjusts its swimming direction based on light stimulus.

The turnover rate for photoactivated adenylate cyclase (PAC) on blue light stimulation has been well characterized [29]. Specifically, studies show that the enzyme’s catalytic activity increases by up to 20-fold upon light activation compared to its dark state. The catalytic constants for the light-dependent conversion of ATP to cAMP are as follows: a turnover number (kcat) of approximately 205 ± 11 min^−1^, which translates to roughly 3.42 molecules per second; a KM (Michaelis constant) of 0.12 ± 0.01 mM; and a catalytic efficiency kcat/KM of 1888 mM^−1^ min^−1^. The experiments have revealed that roughly 6 × 10^7^ cAMP molecules × s^−1^ were produced upon illumination. Taking into account the turnover number, about 2 × 10^7^ PAC molecules need to be employed to achieve this production rate. The photoreceptor is described as a three-dimensional protein crystal with over 100 layers [54]. Each layer is around 7 nm thick. Judging from the TEM images of Rosati et al. (1991) [55], the photoreceptor can be described as an ellipsoid with a length axis of about 3 µm and a short axis of about 2 µm. This corresponds to a volume of about 6.3 × 10^−18^ m^3^. The size of an PAC dimer is in a range of 31.4 Å [29], which is regarded as a sphere for further calculations. Assuming an intermolecular distance of about 90 Å [29], about 1.43 × 10^7^ PAC molecules fit inside the volume of the photoreceptor, which is close to the abovementioned number of PAC molecules necessary to produce the observed amount of cAMP. This means that the number of PAC molecules is not restricted by the size of the photoreceptor.

There are only view reports available, where ATP consumption of a flagellum has been determined [56,57]. Fluorescence-based reporter systems, which employ NADH for ATP regeneration and a marker for ATP consumption, revealed an ATP consumption of about 2.3 × 10^5^–3.2 × 10^5^ ATP molecules per beat in sea urchin sperms, which were embedded in oil droplets [56]. Referring this number for *Euglena gracilis*, ATP consumption at 40 Hz would be in the range of 9.2 × 10^6^–1.28 × 10^7^ ATP molecules per second. Due to the high ATP demand of the flagellum, ATP does not only originate from the cytoplasm, reaching the flagellum via diffusion [58]. In addition, flagella contain a system of glycolytic enzymes [59] and a creatin system [57] and exhibit adenylate-kinase reaction (2 ADP → ADP + AMP) [60]. Probably, the observed ATP consumption of the PAB at illumination may reduce the ATP concentration in the flagellum, reducing the force of the dynein. This may disturb the mechanical equilibrium between the axoneme and the paraflagellar rod, leading to characteristic changes in the flagellar waveform as described in [45]. In further experiments, the specific ATP-producing systems inside the flagellum will be examined in more detail.

## 5. Conclusions

An in vivo cAMP increase in light is dependent on PAC. The speed of synthesis and degradation of cAMP is fast enough to allow oscillations in cAMP, possibly influencing the flagellar beat during one cell rotation. The PAB contains enough PAC molecules to allow the measured increase in cAMP in the observed time.

## Figures and Tables

**Table 1:** Overview of the crRNAs used with their respective target proteins and IDs used in this manuscript; the crRNAs were from Integrated DNA Technologies (IDT).

Knocked-Out Protein	Location of Deletion	crRNA Sequence	ID
PACα	C-terminus	CCAAUGAGGCAUGG	Klone E
PACβ	N-terminus	GGUCACCUUCAUCUA	A
PACβ	N-terminus	UUCAUCUACCUUGUG	B
PACα	N-terminus	GUCACACCCACCAUG	C
PACα	N-terminus	CCCACAAUUCCUUGC	D

## Data Availability

The original contributions presented in this study are included in the article. Further inquiries can be directed to the corresponding author.

## Abbreviations

The following abbreviations are used in this manuscript:

amol	Attomol
cAMP	Cyclic adenosine monophosphate
PAC	Photoactivated adenylyl cyclase
PFR	Paraflagellar rod
